# The Roles of Glycodelin in Cancer Development and Progression

**DOI:** 10.3389/fimmu.2017.01685

**Published:** 2017-11-29

**Authors:** Juan Cui, Yanguo Liu, Xiuwen Wang

**Affiliations:** ^1^Qilu Hospital of Shandong University, Jinan, Shandong, China

**Keywords:** glycodelin, cancer-specific expression, cancer progression, cancer immunity, cancer immunotherapy

## Abstract

Glycodelin is a kind of glycoprotein expressed in secretory endometrium, pregnancy deciduas, and amniotic fluid originally, which is vital for the maintenance of normal human reproductive activities. Recent researches have reported that glycodelin is specifically expressed in various malignancies, including female-specific cancers such as endometrial cancer, ovarian cancer and breast cancer, and non-gender specific cancers including lung cancer, and colon cancer, and glycodelin expression correlates with the diagnosis and prognosis of cancer patients. This review focuses on the expression of glycodelin in different cancers and its role in cancer development and progression. Glycodelin possesses the abilities to regulate cancer cell proliferation, differentiation, and invasion, promote cancer angiogenesis, and modulate the differentiation and function of immune cells including T cells, dendritic cells, monocyte-macrophages, natural killer cells and B cells participating in cancer development. The expression of glycodelin can be regulated by stromal cells, lysophosphatidic acid, histone deacetylase inhibitors, and relaxin. In summary, glycodelin is a promising biomarker for the diagnosis and prognosis of cancer patients, and depending on its distinct immunoregulatory effects, glycodelin can be a prospective target for cancer immunotherapy.

## Introduction of Glycodelin

Glycodelin, also known as pregnancy-associated α2-globulin, placental protein 14, or progesterone associated endometrial protein, is a kind of glycoprotein mainly derived from secretory endometrium, pregnancy deciduas, and amniotic fluid (1, 2). With its distinct glycans and characteristic carbohydrate structure, glycodelin mediates various biological activities in human reproduction and fetomaternal immunity (2, 3). To date, there are four different isoforms of glycodelin identified according to their differences in glycosylation, which are glycodelin A (GdA) mainly from amniotic fluid and pregnancy decidua (4, 5), glycodelin S (GdS) from seminal plasma (6), glycodelin F (GdF) from ovarian follicles (7) and glycodelin C (GdC) from cumulus oophorus (8). The four isoforms of glycodelin exert their distinctive biological functions mainly relying on the protein backbone as well as the glycosylation (3) (Table 1).

**Table 1:** Different isoforms of glycodelin.

Isofrom	Source	Glycosylation	Reproductive functions	Reference
GdA	Amniotic fluid, pregnancy decidua	High sialylation, more fucosylation	Immunoprotection for implantation and placentation, antifertilizing, inhibiting spermatozoa–zona pellucida binding	(4, 5)
GdS	Seminal plasma, seminal vesicles	No sialylated glycans, rich in fucose and mannose	Preventing premature capacitation	(6)
GdF	Ovarian follicles, oviduct	Fucosylated Lewis-x and Lewis-y, more *N*-acetylglucosamine	Inhibiting spermatozoa–zona pellucida, preventing premature acrosome reaction	(7)
GdC	Cumulus oophorus, converted from GdA and GdF	Reacting with specific agglutinins in lectin-binding manner	Stimulating spermatozoa–zona pellucida binding	(8)

*Gd, glycodelin*.

## The Expression of Glycodelin in Cancers

In earlier researches, glycodelin was found mainly from secretory endometrium, pregnancy decidua and amniotic fluid and played an immunoregulatory role in the fetomaternal interface (4). The expression of glycodelin in females is related to some reproductive system diseases such as premature ovarian failure (9), recurrent spontaneous abortion (10), and unexplained infertility (11). To date, more and more studies have reported that glycodelin is expressed in various cancers from female-specific malignancies, such as endometrial cancer, ovarian cancer, and breast cancer, to non-gender specific cancers including lung cancer and colon cancer. And the glycodelin expression in cancers is shown to be associated with diagnosis and prognosis of cancer patients.

### Female-Specific Cancers

#### Endometrial Cancer

Normal secretory endometrium and pregnancy decidua are the main secretory sites for glycodelin in physiological conditions (1), and glycodelin is expressed in normal premenopausal endometrial epithelium tissues but not in postmenopausal epithelium (12). So whether there is an alteration of glycodelin expression in malignant endometrium becomes an early appealing concern for researchers. Glycodelin is detected in cancer tissues of patients with endometrial cancer and MFE-280 cells (13), a cell line derived from a recurrent, poorly differentiated endometrial carcinoma. Moreover, Chatzaki et al. found glycodelin was not present in well-differentiated endometrial adenocarcinoma tissues, and their derived primary malignant cells (12) as well as Ishikawa cells (12, 14), a well-differentiated endometrial adenocarcinoma cell line. It can be speculated that the expression of glycodelin may be correlated with differentiated degree of endometrial carcinoma. Most patients with endometrial cancer are diagnosed at postmenopausal age (15). Compared to normal endometrium, glycodelin mRNA and protein are both overexpressed in endometrial cancer tissues (16, 17). Evaluating glycodelin expression in postmenopausal patients and controls may avoid the disturbance of physiological glycodelin production. As for different histological types, glycodelin is strongly expressed in adenomatous endometrial cancer tissues as wells as moderately expressed in endometrioid endometrial cancer and papillary mucinous endometrial cancer tissues (18). Moreover, higher expression of immunosuppressive isoform GdA is an independent prognostic marker for poorer overall survival in endometrial cancer patients (17) (Table 2).

**Table 2:** Glycodelin expression in endometrial cancer.

Source	Expression of glycodelin	Prognostic relevance of glycodelin expression	Reference
Normal premenopausal endometrium	+		(12)
Normal postmenopausal endometrium	−		(12)
Adenomatous endometrial cancer tissues	++	Poorer overall survival	(17, 18)
Endometrioid endometrial cancer tissues	+		(18)
Papillary mucinous endometrial cancer tissues	+		(18)
MFE-280 endometrial cancer cells	+		(13)
Ishikawa endometrial cancer cells	−		(12, 14)
Plasma of endometrial adenocarcinoma patients (postmenopausal)	High		(19)
Plasma of postmenopausal healthy women	Moderate		(19)
Uterine flushing of endometrial adenocarcinoma patients (postmenopausal)	High		(19)
Uterine flushing of postmenopausal healthy women	Moderate		(19)

*Tissue samples: −, no expression; +, moderate expression; ++, strong expression*.

*Liquid samples: moderate, moderate expression; high, high expression*.

In the plasma of endometrial cancer patients, glycodelin expression is elevated by comparison with healthy controls and also higher than that in ovarian and cervical cancers (16). For the postmenopausal cohort, a higher level of glycodelin is found both in plasma and in uterine flushing of endometrial adenocarcinoma patients than healthy controls without any postmenopausal bleeding (19), which further hints the intimate correlation between glycodelin and endometrial malignant behaviors.

#### Ovarian Cancer

The serum level of glycodelin is reduced in the pregnant women diagnosed as premature ovarian failure than those with normal ovarian functions (9), which implies ovary may play a role in glycodelin production. Glycodelin protein is found both in normal ovary and in ovarian malignant lesions, but when it comes to mRNA detection, ovarian malignant tumors especially serous cystadenocarcinoma and endometrioid adenocarcinoma exhibit strong expression while normal ovary is glycodelin-negative (20), indicating that ovarian cancer abnormally produces glycodelin whereas normal ovary may absorb glycodelin or just offer a site for function. It could be concluded that glycodelin would participate in the malignant transformation in normal tissue environment.

Glycodelin expression varies with different histopathological subtypes of ovarian cancers, which may involve variant malignant evolution processes. Glycodelin is expressed in epithelial ovarian cancer tissues including serous, mucinous, endometrioid, and clear cell cancer types and non-epithelial granulose cell ovarian cancer (18, 21, 22). Among the above histopathological subtypes, glycodelin expression in serous ovarian carcinoma and GdA in mucinous ovarian carcinoma are the intensest (21, 22) (Table 3).

**Table 3:** Glycodelin expression in ovarian cancer.

Source	Expression of glycodelin	Prognostic relevance of glycodelin expression	Reference
Normal ovary tissues (preimplantation or postmenopausal)	−		(20)
Advanced stage epithelial ovarian cancer tissues (stage III and IV)	++	Shorter overall survival, 5-year survival and recurrence-free survival	(22)
Serous ovarian cancer tissues	++		(18, 21, 23)
Grade 1	++	Longer 5- and 10-year overall survival	
Grade 2	+		
Grade 3	+		
Stage I	++		
Stage II	++		
Stage III	+	Longer 5-year and 10-year overall survival	
Stage IV	+		
Mucinous ovarian cancer tissues	++		(18, 21)
Endometrioid ovarian cancer tissues	+		(18, 21)
Clear cell ovarian cancer tissues	+		(18, 21)
Granulose cell ovarian cancer tissues	+		(21)
Cystic fluids, serum of ovarian cancer patients	High		(16, 21, 24, 25)
Cystic fluids, serum of benign ovarian lesion patients	Moderate		
Cystic fluids, serum of healthy women	Low		

*Tissue samples: −, no expression; +, moderate expression; ++ , strong expression*.

*Liquid samples: low, low expression; moderate, moderate expression; high, high expression*.

In virtue of its specific expression in ovarian cancer, glycodelin is expected to be a useful biomarker for the diagnosis of ovarian cancer. The expression of glycodelin increases significantly in serum, cystic fluids and tissues of patients with ovarian cancer in comparison with benign ovarian lesions and healthy controls (16, 18, 21, 24–26). In contrast with CA125, the well-applied clinical serum biomarker for ovarian cancer, glycodelin also performs highly diagnostic sensitivity for early ovarian cancer (27). Along with other biomarkers such as CA125, MMP7, or HE4, glycodelin prominently improves the sensitivity and specificity for early stage ovarian cancer diagnosis and recurrence monitoring (25). Others seem to provide controversial results to query the biomarker role of glycodelin. Richter et al. investigated GdA expression in tissues of ovarian cancer patients and control patients and no significant difference was found. There were only 27 cases in the ovarian cancer group and the controls included 48 patients with uterus myomatosus, endometriosis, cervical, endometrial carcinoma, breast cancer, bladder carcinoma, or ovarian metastases of a colon carcinoma. Samples from neither normal ovary nor benign ovarian lesions were investigated in the study (28). Riittinen et al. did not find any significant dissimilarity of glycodelin expression in cysts fluids among benign, borderline and malignant ovarian lesions (29). Scholz et al. revealed glycodelin level in patients’ serum was higher in benign ovarian tumors than in ovarian cancers (22). Benign or borderline ovarian tumors can be considered as precursor lesions of ovarian cancer and the inflammation of ovarian epithelium can increase the risk of malignant transformation (30, 31). Glycodelin can mediate the immunosuppression to various inflammatory cells and also participates in inflammation regulation in precursor lesions of ovarian cancer, which may subsequently fluctuate the expression of glycodelin (32).

Apart from diagnostic significance, glycodelin is revealed to have potent correlation with survival of ovarian cancer patients. Positive expression of GdA in epithelial ovarian cancer tissues is demonstrated to independently predict poorer prognosis of advanced stage (FIGO stage III and IV) patients who have shorter overall survival, 5-year survival and recurrence free survival (22). However, in others’ findings, glycodelin expression hints better cancer differentiation and earlier disease stage in ovarian serous carcinoma, one of the most common epithelial ovarian cancer subtypes. Glycodelin is expressed in the cytoplasm of ovarian serous carcinoma with intenser staining in grade 1 (well differentiation) than grade 2 or 3 (poor-differentiation) carcinoma and in FIGO stage I (early-stage) than stage III or IV (advanced-stage) carcinoma (23). Patients with positive glycodelin expression possess longer 5- and 10-year overall survival than those without glycodelin expression, especially notable for the cohort of grade 1 carcinoma or stage III disease in spite of glycodelin not as an independent variable (23). Tsviliana et al. presented GdA expression was elevated in grade 1 than grade 2 tissues and in FIGO stages I–II than stages III–IV ovarian cancer. Although the eligible samples cover serous, endometrioid, mucinous, and clear cell ovarian cancers, the grading and staging relevance to different histopathological subtypes is not further investigated (33) (Table 3). The controversial results of glycodelin expression pointing to diverse differentiation grades, stages and survival of ovarian cancer patients, may be attributed to the heterogeneity of ovarian cancers. The heterogeneity results in discrepancies in the search for biomarkers and targeted treatments of ovarian cancers, which varies with the histological types, stages, grades of ovarian cancer, patient’s age, or genetic background. Aberrant epithelial differentiation leads to the malignant transformation of ovarian cancer which is regulated by multiple genes and mechanisms in cancer microenvironment. Whether glycodelin interacts with cancer microenvironment in ovarian cancer progression is worthy of exploration.

#### Breast Cancer

Glycodelin expression in breast cancer and its correlation with patient characteristics are extensively investigated. In the familial non-BRCA1/2 mutated breast cancer patients with strong family history, glycodelin is more frequently expressed in the tissues with malignant features including lymph node metastasis, positive HER2, and negative ER, and PR status. Besides, glycodelin positive-expression independently predicts an increased metastasis risk and indicates poorer 10-year cancer-specific and 5-year metastasis-free survival (34), which suggests that glycodelin can be a potent factor to predict incremental mortality risk for these patients. In the subgroup analysis for stage II and III breast adenocarcinomas mostly with lymph node metastasis, glycodelin is overexpressed in cancer tissues of patients characterized by younger age, premenopausal status and positive HER2 expression. No statistical correlation is observed between glycodelin and survivin expression and glycodelin cannot predict overall survival and disease free survival for these patients (35). For sporadic breast cancer patients, glycodelin manifests denser staining in the cancer tissues with less aggressive features including low proliferation rate, well-differentiated histological grade and high cyclin D1 expression, while no relation is found between glycodelin expression and patient survival (34). Besides, glycodelin is expressed more in the tissues of breast cancer *in situ* and invasive breast cancer without lymph node metastases than those with lymph node metastases (18, 36, 37), but more samples should be recruited into further studies. While in the 121 invasive cancer specimens from lobular and ductal breast cancers glycodelin expression specifically reduces upon dedifferentiation grading (G1 to G3) of cancer tissues, it does not correlate with axillary lymph node metastasis and steroid receptor (ER and PR) status (38) (Table 4).

**Table 4:** Glycodelin expression in breast cancer.

Source	Expression of glycodelin	Prognostic relevance of glycodelin expression	Reference
Normal breast tissues	−		(39)
Familial non-BRCA1/2 breast cancer tissues with strong family history	+	Poorer 10-year cancer-specific and 5-year metastasis-free survival	(34)
Positive HER2	++		
Negative ER and PR status	++		
Lymph node metastasis	++		
Stage II–III breast cancer tissues (mostly with lymph node metastasis)	+		(35)
Younger age	++		
Premenopausal status	++		
Positive HER2 expression	++		
Sporadic breast cancer tissues	+		(34)
Low proliferation rate	++		
Well-differentiated histological grade	++		
High cyclin D1 expression	++		
Breast cancer *in situ* tissues	++		(18, 36, 37)
Invasive breast cancer tissues without lymph node metastases	++		
Invasive breast cancer tissues with lymph node metastases	+		
Invasive breast cancer tissues with recurrence and metastases	+		
Lobular and ductal breast cancer tissues	+		(38)
Grade 1	+++		
Grade 2	++		
Grade 3	+		

*Tissue samples: −, no expression; +, moderate expression; ++, strong expression; +++, extra strong expression*.

Based on the above, it can be speculated that the expression level or predictive effect of glycodelin for breast cancer may vary with eligible subjects, sample size, relevant experimental methods and operating conditions, and whether glycodelin is involved in the progression of different subtypes of breast cancer deserves further validation.

#### Cervical Cancer

In comparison with healthy adult control group, there is an elevated plasma level of glycodelin in cervical cancer patients (16). Besides, the immunosuppressive isoform GdA is expressed in all histologically normal, dysplastic and malignant squamous epithelium from 14 uterine cervical sections (40). A larger sample size is needed to explore cervical cancer-related specific expression of glycodelin.

### Non-Gender Specific Cancers

#### Lung Cancer

Both mRNA transcripts examination and protein immunohistochemical staining demonstrate increased glycodelin expression in lung adenocarcinoma and lung squamous carcinoma over normal lung tissues and its expression in lung adenocarcinoma is higher than that in lung squamous carcinoma (41, 42). Kunert-Keil et al. also found glycodelin was overexpressed in lung adenocarcinoma and lung squamous carcinoma as well as lung metastases of colon cancer compared to normal lung tissues (43) (Table 5).

**Table 5:** Glycodelin expression in other cancers.

Source	Expression of glycodelin	Prognostic relevance of glycodelin expression	Reference
Normal lung tissues	−		(41, 43)
Lung adenocarcinoma tissues	++/+		
Lung squamous carcinoma tissues	+		
Lung metastases of colonic adenocarcinoma tissues	+		
Serum of female NSCLC patients	High	Poorer overall survival	(41)
Serum of women with benign lung diseases	Moderate		
Serum of metastatic colorectal cancer	High		(44)
Effective therapy	50% drop or more		
In stable status	50% drop or more		
Serum of healthy adults	Moderate		
Serum of MPM patients	High	Poorer overall survival	(45)
Serum of patients with benign lung diseases	Moderate		
Male MPM tissues	+	Improved overall survival	(45)
Male benign lung disease tissues	−		
Melanoma tissues	+		(42, 46, 47)
Normal human skin tissues	−		
Biphasic synovial sarcomas tissues(11/11)	+++		(48)
Monophasic sarcomas tissues (1/7)	**+**		

*Tissue samples: −, no expression; +, moderate expression; ++, strong expression; +++, extra strong expression*.

*Liquid samples: moderate, moderate expression; high, high expression*.

As a kind of secretory protein, glycodelin detected in the sera of non-small cell lung cancer (NSCLC) patients presents obviously a higher level than in the cohort of benign lung diseases. Female NSCLC patients with lymph node metastasis secrete more glycodelin than those without lymph node metastasis. When the limited cancer is excised completely by surgery, the serum glycodelin reduces significantly, and it increases following the recurrence or metastasis (41). Higher glycodelin expression in female NSCLC patients indicates a poorer overall survival rate (41). In the case report from the above included patients, the expression of glycodelin in a 65-year-old male patient diagnosed as lung adenocarcinoma is elevated significantly along with cancer progression of recurrence and metastasis, presenting a concomitant change with the serial cancer status from initial diagnosis, lobectomy, chemotherapy, cancer progression to cancer death (49). The evidence implies that glycodelin can be a potent biomarker for NSCLC diagnosis, monitoring, and prognosis.

#### Colorectal Cancer

In contrast with healthy controls, serum level of glycodelin increases significantly in the patients with metastatic colorectal cancer before any treatment, and when the patients experience effective therapy or are in stable status, the serum glycodelin also shows a more than 50% drop (44) (Table 5).

#### Malignant Pleural Mesothelioma (MPM)

Glycodelin is revealed to be an available serum biomarker for MPM. The serum level of glycodelin is found increased in patients with MPM in comparison to those with benign lung diseases. Higher serum level of glycodelin is related to worse therapeutic sensitivity and poorer overall survival in MPM patients. Together with soluble mesothelin-related peptide, a useful biomarker for MPM, serum glycodelin improves the prognostic efficiency for MPM. Both total glycodelin and the immunoregulatory isoform GdA are strongly expressed in MPM tumor tissues, but in survival analyzes, overexpressed GdA in males not females indicates an improved overall survival, which may be related to the inflammatory status between the pleural layers (45) (Table 5).

#### Melanoma

Glycodelin is overexpressed in melanoma than normal skin tissues (42), especially in thick primary and metastatic melanomas and their derived cell lines with great invasive properties (46). In melanoma and derived daughter cell lines, overexpressed glycodelin also shows a strongly positive correlation with microphthalmia-associated transcription factor (MITF) expression, a key regulatory gene in melanoma oncogenesis and progression (47). Whether the melanoma-specific expression of glycodelin has diagnostic and prognostic values for the patients would be a valuable research point (Table 5).

#### Biphasic Synovia Sarcomas

Glycodelin is highly expressed in all biphasic synovial sarcomas (11/11) with epithelial glandular differentiation but slightly expressed in monophasic sarcomas (1/7) (48), regarding the monoclonal origin of synovial sarcomas, which hints that glycodelin may play a role in the malignant transformation in synovial sarcomas (Table 5).

## The Effects of Glycodelin in Cancer Development and Progression

### Cell Proliferation, Differentiation, and Invasion

Glycodelin is implicated owning the potential to facilitate malignant behaviors of cancer cells. When glycodelin is silenced by siRNA, melanoma and NSCLC cells exhibit a weaker ability to proliferate, invade, and metastasize and shows attenuated tumorigenesis in mice xenograft model (41, 46, 47). MITF promotes glycodelin expression of melanoma cells and the crosstalk between MITF and glycodelin may regulate melanoma progression (47).

However, in other cancers glycodelin may have opposite roles in cancer progression. When glycodelin is overexpressed by cDNA transfection, the cell proliferation is subdued in endometrial cancer HEC-1B and Ishikawa cells and breast cancer MCF-7 cells (50–52). Overexpressed glycodelin is also accompanied by other alterations in the cells. The morphology of HEC-1B cells is changed to compact spherical structures without acini as well as downregulated expression of cancer-related genes MUC1 and Bcl-X_L_ (50). The cell cycle of Ishikawa cells is hindered to S phase with increased expression of cyclin-dependent kinase (CDK) inhibitors p21, p27, and p16 (51). MCF-7 cells show better differentiation features with well-organized glandular structures and smaller tumor formation in the mice tumor xenograft. The gene expression profile is also altered in MCF-7 cells *in vitro*. Oncogenes like CDK2 and MMP1 are downregulated and suppressive genes such as caveolin-1 and FGF2 are upregulated. E-cadherin and cytokeratins 8 and 18 are also increased as well as more membranous β-catenin expression (52, 53). When the transfected MCF-7 cells are treated by PMA, which can activate the PKCδ activity to induce cell migration and invasion, the control cells show twofold increase of migration ability than transfected cells (54).

The roles of glycodelin in cell proliferation, differentiation, and invasion seem to be contradictory among different cancers. It may be explained partially by that glycodelin may participate in multiple carcinogenesis processes in different cancers with diverse malignant degrees. Besides, different antigenic epitopes of glycodelin applied in the experiments comprise liner, conformational, or specific glycosylation patterns (46), so the detection of intact wild-type glycodelin would be more convincing.

### Angiogenesis

In addition to its expression in cancer cells, glycodelin also presents in the endothelium of cancer blood vessels (16, 55). Human umbilical cord vein endothelial cells (HUVECs) can accumulate glycodelin *in vitro* (56). Treatment with glycodelin promotes proliferation, migration, and tube formation of HUVECs and facilitates angiogenesis, which is mediated by the angiogenic factor VEGF. Moreover, glycodelin increases VEGF expression in various cancers including endometrial cancer (RL-95 cells), ovarian adenocarcinoma (OVCAR-3 cells), and breast cancer (MCF-7 and MDA-MB-231 cells) (57). In the process to promote angiogenesis, glycodelin induces more expression of β-catenin in the perimembrane areas of HUVECs in a dose-dependent manner (58), and upregulated β-catenin can reinforce the intercellular adhesion of HUVECs to facilitate angiogenesis. Solid tumors relies on angiogenesis to spread, invade and metastasize, facilitated by various angiogenic activators like VEGF and bFGF (59). Thus combined with glycodelin-blocking therapy, the efficacy of antiangiogenic agents might be improved.

### The Immunoregulatory Effects of Glycodelin

**Figure 1:**
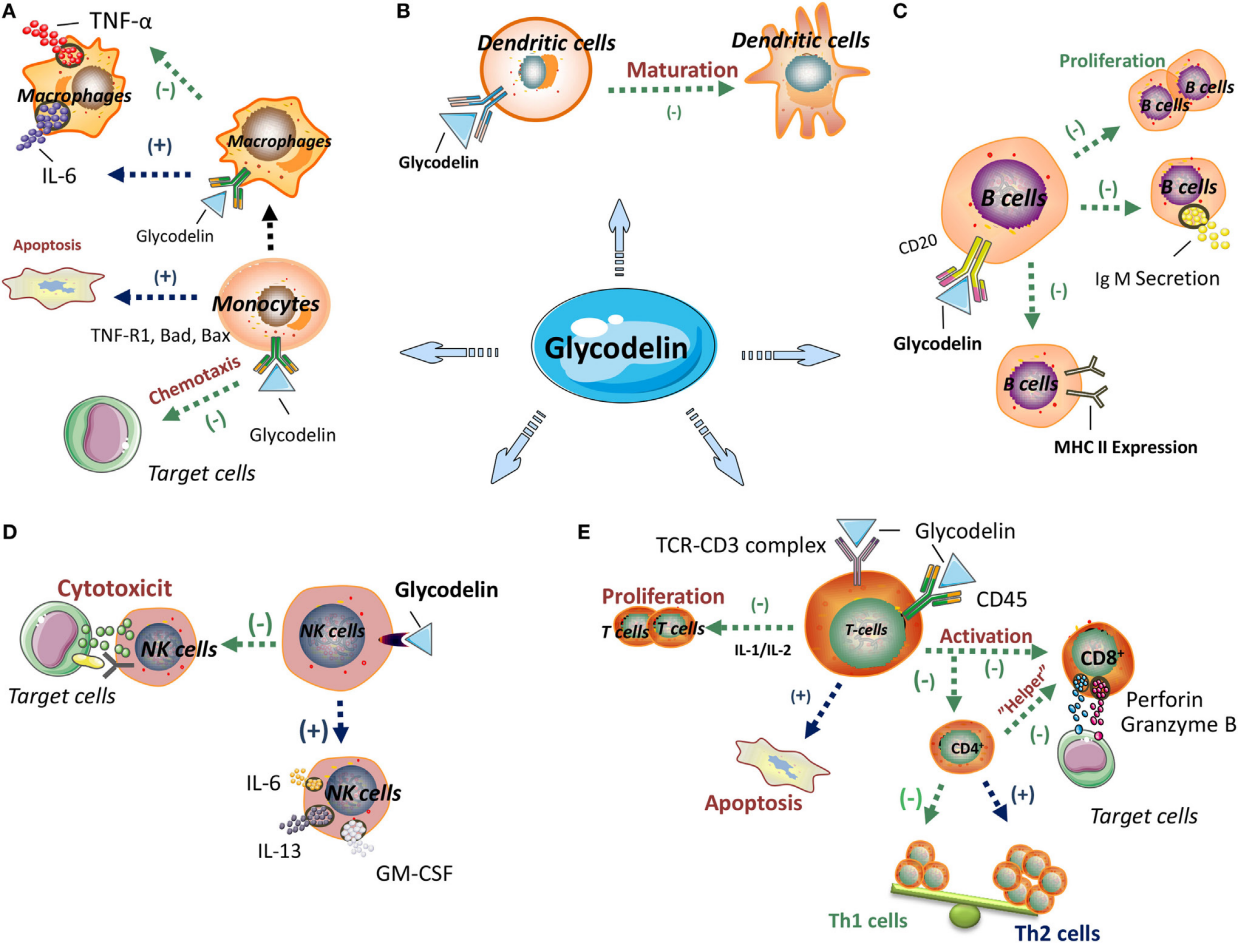
The immunoregulatory effects of glycodelin. Glycodelin exerts immunoregulatory effects on multiple immune cells including monocyte-macrophages, dendritic cells (DCs), B cells, natural killer (NK) cells and T cells. **(A)** Glycodelin suppresses the monocyte chemotaxis and facilitates its apoptosis, and reduces TNF-α secretion by macrophages, which further influences innate immunity. **(B)** Glycodelin impairs the maturation of DCs and inhibits their immunogenic T-cell stimulatory capacity. **(C)** Glycodelin inhibits the proliferation, IgM secretion and MHC class II expression of stimulated B cells to regulate humoral immunity. **(D)** Glycodelin decreases the cytotoxicity of NK cells and upregulates its secretion of IL-6, IL-13, and GM-CSF which results in attenuated lethal effect against target cells. **(E)** Glycodelin skews the polarization of naive CD4+ T cells toward T helper type 2 (Th2) but not Th1 subsets and subdues the cytotoxic effects of CD8+ T cells. (−) Inhibition; (+) promotion. The elements in the figure, including signs or icons for cells and molecules, were derived for Servier Medical Art^1^ and ScienceSlides^2^ with permission.

^1^https://www.servier.hk/content/servier-medical-art.

^2^http://www.scienceslides.com/.

Glycodelin has been illustrated to exert immunoregulatory effects to multiple immune cells including T cells, dendritic cells (DCs), monocyte-macrophages, natural killer (NK) cells, and B cells (Figure 1).

#### T cells

In early researches, the significant immunosuppressive effects of glycodelin were reflected in proliferation inhibition and apoptosis induction of stimulated lymphocytes (60–63) and as the researches moving on, the interaction between glycodelin and specific subtypes of lymphocytes is further refined. Glycodelin exerts different effects on T helper type 1 (Th1) and Th2 subsets. Glycodelin can inhibit activities of both Th1 and Th2 cells, but the inhibition to Th1 cells is more significant (64). Moreover, glycodelin skews the polarization of naive CD4+ T cells toward Th2 subsets during T cell priming, *via* suppressing Th1 cytokines (IFN-γ and IL-2) secretion and chemokine receptor CXCR3 expression and impairing downregulation of GATA-3 expression, which are all vital for the development and activation of Th1 cells (64). Glycodelin exhibits equal adhesion activity to Th1 and Th2 cells, but increases the cell death rate, upregulates Fas expression, enhances caspase-3 and -9 activities and inhibits activation of phosphorylated ERK in Th1 cells, not in Th2 cells (65). With regard to CD8+ T cells, glycodelin impairs the cytotoxic effects of alloactivated CD8+ T cells toward target cells, involving attenuated expression of granzyme B and perforin, two important cytolytic effector molecules, and subdued degranulation of cytolytic vesicles. Despite inhibiting the proliferation of CD8+ T cells, glycodelin does not affect the apoptosis of CD8+ T cells (66), which may be attributed to the different regulatory mechanisms between proliferation and apoptosis in CD8+ T cells (Figure 1).

The immunoregulatory functions of glycodelin may rely on various molecules and signal transduction pathways. Glycodelin engages in the early TCR-CD3 signal transduction for the activation and responses of T cells, mainly including several mechanisms as follows. (a) Glycodelin abrogates TCR-induced Ca^2+^ fluxes for the early TCR-CD3 signal events (67). (b) Different from other T-cell inhibitors such as CSA, which inhibits Ca^2+^-dependent phosphatase (calcineurin) to weaken the activation signaling pathway independent of TCR engagement, glycodelin works as a “rheostat” to elevate the threshold of TCR activation with higher intensity of stimulus and change corresponding cytokine expression profile, but not to block T-cell signal transduction directly (68). (c) Glycodelin can localize in the APC-T cell contact sites of TCR triggering to impede T cell activation (69), instead of indirectly inhibiting costimulation of accessory cells such as activated monocytes (67). (d) Glycodelin binding to intact CD45 of T cells dampens TCR-CD3 signaling transduction (70, 71).

By addition of glycodelin, IL-2/IL-2R signaling in T cells is disturbed owing to decreased expression of IL-2R on cell surface, which subsequently arrests proliferation of T cells and impairs immune responses including attenuated cytotoxicity of CD8+ T cells and proapoptosis in CD4+ “helper” cells (72). Glycodelin also triggers mitochondrial stress directly leading to the apoptosis of T cells independent of TCR and CD45 signaling (73). As a kind of glycoprotein, glycodelin accelerates the apoptosis of T cells by selectively combining N-linked glycans on T cell surface glycoproteins (74). Activated T cells expose more galactose compared to naive T cells (74) and only activated T cells can be induced apoptosis by glycodelin (63). On the contrary, Ish-Shalom et al. stressed N-glycosylation and oligosaccharide side chains of glycodelin were not indispensable for the binding to T cell surface (71). Glycodelin exerts its proapoptotic role dependent on the protein backbone rather than the oligosaccharides of glycosylation (75). It is mapped that the stretch of amino acid sequence of glycodelin between Met24 and Leu105 is necessary to inhibit proliferation and induce apoptosis^2^ of T cells (76).

Diverse isoforms of glycodelin have different immunomodulating effects on T cells (77–79). Among the four isoforms, both GdA and GdF inhibit the immunocompetence of T cells through proliferation arrest, apoptosis induction and IL-2 downregulation, while GdS and GdC do not exhibit any immunosuppressive effects (79). Different from the GdA with proapoptosis property, GdS dose not perform any apoptosis-induced ability in T cells, which is due to the glycans of glycosylation presented in GdA and sialic acid residues absent in GdS (77, 78). GdS and GdC, as well as deglycosylated glycodelin, cannot influence the Th1/Th2 polarization from CD4+ T cells (65).

The glycodelin derived from ovarian cancerous ascites (80) and melanoma cells (81) shows the distinct negative regulation of T lymphocytes with diminished secretion of IL-2 and IFN-γ, arrested proliferation or cytotoxic ability and elevated apoptosis (81). Glycodelin also shows a potent immunomodulatory effect in NSCLC. When glycodelin is silenced in NSCLC cells, several immune system modulators such as programmed death-1 ligand (PD-L1) as well as chemokines CXCL5 and CXCL16 are remarkably upregulated (41). From the above, glycodelin reveals vital immunoregulatory effects in T cell proliferation, differentiation, and functions, which can be inferred that glycodelin could act as the mediator between cancer progression and T cell immunity.

#### Dendritic Cells

Glycodelin A impairs the maturation of DCs from peripheral blood mononuclear cells (PBMCs) (82). When pretreated with GdA, immature DCs cannot shift into complete mature phenotype. In comparison with fully matured DCs, the tolerogenic effects of immature DCs consist of discriminated expression of costimulatory molecules CD83 and CD86, unchanged expression of MCH-II and DC-SIGN, persisted endopinocytotic activity, increased IL-10 production and reduced lymphoproliferative activity (82). The glycodelin protein purified from the malignant ascites of ovarian cancer patients, also hinders the maturation of DCs with the tolerogenic phenotype and functionality (80), which suggests glycodelin can form an immunosuppressive microenvironment in cancer progression (Figure 1).

#### Monocyte-Macrophages

Glycodelin suppresses the chemotaxis ability of human monocytes (U937 cells) in a dose-dependent manner (83). Unlike the interaction of glycodelin with T cells, neither glycosylation nor sialylation of glycodelin plays a part in impairing chemotaxis ability for monocytes (83, 84). Recombinant glycodelin distinctly binds with a specific protein receptor in CD14+ monocytes, but the corresponding receptor does not exist in the surface of CD3+ T cells or CD20+ B cells (85).

In the mechanism exploration that glycodelin facilitates monocytes to apoptosis, glycodelin regulates the expression of apoptosis-related genes in monocytes including decreasing antiapoptotic genes Bcl-2A1 and APRIL and increasing proapoptotic genes TNF-R1, Bad, and Bax. Glycodelin treatment in monocytes also arrests transcription factor NF-κB and activates caspase-8, -2, and -3 and the involved signaling directs the mitochondrial apoptotic pathway independent of MAP kinases (JNK and p38) and caspase 2 (84, 86). In the comparison of effects by glycodelin between T lymphocytes and monocytes, glycodelin inhibits the proliferation both in T cell lines (Jurkat cells) and monocytic cell line (U937 cells), but only T cells are induced apoptosis and no apoptosis is obtained in monocytes. It may be on account of caspase 3, an vital enzyme in the apoptosis of mammalian cells, elevated in T cells but without change in monocytes (63) (Figure 1).

Glycodelin A reduces TNF-α secretion of macrophages derived from monocytes (87) but does not influence their phagocytic ability (86). Despite without changing the viability, cell death, or phagocytosis of monocytes/macrophages, GdA promotes the secretion of IL-6 (87, 88) by interacting with L-selectin in monocytes/macrophages to activate phosphorylated ERK. Subsequently, the induced IL-6 inhibits IFN-γ expression of Th cells, which results in the activating response of CD4+ T cells skewing toward Th2 subsets (88).

#### NK Cells

In spite of little or absent expression in peripheral blood NK cells, glycodelin is selectively expressed in decidual NK cells (89). When exposed to glycodelin in considerable concentration range, the cytotoxicity of NK cells against target cells is impaired directly although the binding ability of NK cells to target cells is retained, without need for prostaglandin induction (90). Lee et al. indicated (91) GdA did not have modulatory roles in the viability, cell death, cytotoxicity and phenotype of NK cells from the peripheral blood, but their cytokine secretion of IL-6, IL-13, and GM-CSF was upregulated by GdA treatment (Figure 1).

#### B Cells

Glycodelin also regulates humoral immunity. Glycodelin suppresses the proliferation, IgM secretion and MHC class II expression of stimulated B cells, without influencing other surface molecules, such as CD69 and CD86. And the inhibition is not related to BCR triggering (26). In a carbohydrate-dependent manner (26), the oligosaccharide chains in the N-glycosylation of glycodelin specifically combine with CD22 in B cells to exert its immunosuppressive functions (92) (Figure 1).

## The Regulators of Glycodelin in Cancers

Glycodelin expression in cancers is regulated by diverse cells and molecules. Stromal paracrine signals may play a role in the regulation of glycodelin expression. The stromal cells in endometrium induce the production of glycodelin both in endometrial cancer cells (Ishikawa cells) (14) and in normal endometrial epithelial cells (93). It can be postulated that glycodelin may interact with cancer microenvironment to modulate cancer development.

Lysophosphatidic acid (LPA) is overexpressed in many malignancies especially gynecologic cancers (94, 95). LPA can increase glycodelin expression in cervical cancer (Hela cells), endometrial cancer (RL-95 cells), and ovarian cancer (OVCAR-3 cells) but not in breast cancer (MDA-MB-231 cells) (96), which further implies that glycodelin may be involved in LPA-induced signaling to participate in cancer development and progression.

As a kind of promising anticancer drug, histone deacetylase inhibitors (HDACIs) regulate cancer development and the expression of cancer-related genes (97). Glycodelin is upregulated by HDACIs, trichostatin A, and suberoyl anilide hydroxamic acid, which further speeds the migration of Ishikawa endometrial adenocarcinoma cells (98) but promotes cell differentiation to the normal epithelium characteristics (99), which generates a dilemma in the clinical use of HDACIs for anticancer treatment.

Relaxin, a mediator to promote the progression of various cancers such as endometrial cancer and breast cancer, activates glycodelin expression in endometrial glandular epithelial cells *in vitro*. The serum expression profiles of glycodelin and relaxin are closely correlative and glycodelin expression in human serum also increases following relaxin administration *in vivo* (100, 101). Taylor et al. further elucidated that relaxin inhibited progestin-stimulated activation of glycodelin in Ishikawa cells rather than regulating glycodelin production directly (102).

## Conclusion

Glycodelin is extensively known as a kind of glycoprotein whose glycans and protein structures mediate various actions in human reproductive activities. There are four known isoforms of glycodelin to date, GdA, GdS, GdF, and GdC. Due to the specific glycosylation, GdA rather than other isoforms occupies the majority of glycodelin, and in early researches it is proved to play an indispensable role in fetomaternal immunity. Thus recent researches pay more attention to the role of GdA in cancer development and progression. Whether other isoforms of glycodelin also participate in cancer development and progression need further investigations.

Glycodelin expression is closely related to the diagnosis and prognosis of cancer patients; and therefore, it could be a potential biomarker for early diagnosis and recurrence monitoring of cancer patients. Dependent on the abilities to regulate cell proliferation, differentiation, and invasion and promote angiogenesis, glycodelin can remodel the cancer microenvironment and adjust the expression of cancer-related genes and the presentation of cell surface molecules. The specific expression of glycodelin in cancers may provide a general mechanism to facilitate cancer development and progression.

The present researches have illustrated the immunologic effects of glycodelin to multiple immune cells, and the investigated immune cells are mostly isolated from PBMC, which can be extended to the correlation between glycodelin and cancer immunity. Additionally, glycodelin expressed in the normal hematopoietic cells, including megakaryocytic lineage and erythroid precursors (103, 104), indicates that glycodelin is also involved in the human entire immune activities not just in the local reproductive immunity.

However, there are some limitations that cannot be ignored. First, some controversial results are presented about prognostic value of glycodelin in cancer patients. These discrepancies may rely on different detection methods or reagents. It would be of great significance to produce quality antibodies and establish considerable standards for glycodelin detection. Second, parts of the reported studies are of relatively small sample size. Studies with large sample size or systemic meta-analysis on the correlation of glycodelin expression with cancer patient prognosis need be further explored. Third, the role of glycodelin A in cancer development and progression is well investigated and mainly discussed in the review, but the roles of other glycodelin isoforms, including GdS, GdF, and GdC, have not been fully revealed due to various limitations. Finally, as a kind of reproduction-related glycoprotein, normal secretory endometrium of females is a main source of glycodelin in physiological conditions, so glycodelin is indicated of more significance in female-specific cancers such as endometrial cancer, ovarian cancer, and breast cancer. Besides, in non-gender specific cancers such as lung cancer, female NSCLC patients with lymph node metastases have higher glycodelin secretion and higher glycodelin expression in female NSCLC patients indicates a poorer overall survival rate (41). Thus glycodelin may be of greater importance for female patients.

In the past decades, immunotherapy has become the main breakthrough in cancer therapy by overcoming cancer immune evasion (105). The current clinical success, immune checkpoint inhibitors, is aimed to unleash antitumor immunity to eliminate cancer cells by specific blocking antibodies to cytotoxic T lymphocyte antigen-4 (CTLA-4) and programmed death-1 (PD-1), two inhibitory receptors in T cells to limit lymphocyte activation (106). It is well desired to investigate that whether glycodelin participates in the regulation of negative costimulatory signaling pathways, like CTLA-4/B-7 and PD-L1/PD-1 and other T cell subsets such as Th17 and regulatory T cells. When PD-L1/PD-1 pathway is blocked in cancer, the polarization of effector T cells is skewed in the balance of Th1/Th17 (107). Treg cells can accelerate the immune privilege in cancer (108) and if glycodelin produces immunoregulatory effects to Treg cells, it will provide further evidence for how glycodelin participates in cancer immunity.

## Author Contributions

JC, YL, and XW conceived the idea, and JC and YL wrote the article. All authors reviewed the manuscript.

## Conflict of Interest Statement

The authors declare that the research was conducted in the absence of any commercial or financial relationships that could be construed as a potential conflict of interest.

## References

[1] Bell SC Patel S Hales MW Kirwan PH Drife JO. Immunochemical detection and characterization of pregnancy-associated endometrial alpha 1- and alpha 2-globulins secreted by human endometrium and decidua. J Reprod Fertil (1985) 74(1):261–70. 10.1530/jrf.0.0740261 2410613

[2] Seppala M Koistinen H Koistinen R. Glycodelins. Trends Endocrinol Metab (2001) 12(3):111–7. 10.1016/S1043-2760(00)00365-9 11306335

[3] Seppala M Koistinen H Koistinen R Chiu PC Yeung WS. Glycosylation related actions of glycodelin: gamete, cumulus cell, immune cell and clinical associations. Hum Reprod Update (2007) 13(3):275–87. 10.1093/humupd/dmm004 17329396

[4] Julkunen M Rutanen EM Koskimies A Ranta T Bohn H Seppala M. Distribution of placental protein 14 in tissues and body fluids during pregnancy. Br J Obstet Gynaecol (1985) 92(11):1145–51. 10.1111/j.1471-0528.1985.tb03027.x 4063232

[5] Koistinen H Easton RL Chiu PC Chalabi S Halttunen M Dell A Differences in glycosylation and sperm-egg binding inhibition of pregnancy-related glycodelin. Biol Reprod (2003) 69(5):1545–51. 10.1095/biolreprod.103.017830 12826581

[6] Koistinen H Koistinen R Dell A Morris HR Easton RL Patankar MS Glycodelin from seminal plasma is a differentially glycosylated form of contraceptive glycodelin-A. Mol Hum Reprod (1996) 2(10):759–65. 10.1093/molehr/2.10.759 9239694

[7] Tse JY Chiu PC Lee KF Seppala M Koistinen H Koistinen R The synthesis and fate of glycodelin in human ovary during folliculogenesis. Mol Hum Reprod (2002) 8(2):142–8. 10.1093/molehr/8.2.142 11818517

[8] Chiu PC Chung MK Koistinen R Koistinen H Seppala M Ho PC Cumulus oophorus-associated glycodelin-C displaces sperm-bound glycodelin-A and -F and stimulates spermatozoa-zona pellucida binding. J Biol Chem (2007) 282(8):5378–88. 10.1074/jbc.M607482200 17192260

[9] Critchley HO Chard T Olajide F Davies MC Hughes S Wang HS Role of the ovary in the synthesis of placental protein-14. J Clin Endocrinol Metab (1992) 75(1):97–100. 10.1210/jcem.75.1.1619035 1619035

[10] Tulppala M Julkunen M Tiitinen A Stenman UH Seppala M. Habitual abortion is accompanied by low serum levels of placental protein 14 in the luteal phase of the fertile cycle. Fertil Steril (1995) 63(4):792–5. 10.1016/S0015-0282(16)57483-4 7890064

[11] Li TC Dalton C Hunjan KS Warren MA Bolton AE. The correlation of placental protein 14 concentrations in uterine flushing and endometrial morphology in the peri-implantation period. Hum Reprod (1993) 8(11):1923–7. 10.1093/oxfordjournals.humrep.a137961 8288761

[12] Chatzaki E Gallagher CJ Iles RK Ind TE Nouri AM Bax CM Characterisation of the differential expression of marker antigens by normal and malignant endometrial epithelium. Br J Cancer (1994) 69(6):1010–4. 10.1038/bjc.1994.198 7515261 PMC1969425

[13] Hackenberg R Loos S Nia AH Kunzmann R Schulz KD. Expression of placental protein 14 by the new endometrial cancer cell line MFE-280 in vitro and by endometrial carcinomas in vivo. Anticancer Res (1998) 18(2A):1153–8. 9615781

[14] Arnold JT Lessey BA Seppala M Kaufman DG. Effect of normal endometrial stroma on growth and differentiation in Ishikawa endometrial adenocarcinoma cells. Cancer Res (2002) 62(1):79–88. 11782363

[15] Buchanan EM Weinstein LC Hillson C. Endometrial cancer. Am Fam Physician (2009) 80(10):1075–80. 19904892

[16] Horowitz IR Cho C Song M Flowers LC Santanam N Parthasarathy S Increased glycodelin levels in gynecological malignancies. Int J Gynecol Cancer (2001) 11(3):173–9. 10.1046/j.1525-1438.2001.01017.x 11437921

[17] Lenhard M Heublein S Kunert-Keil C Vrekoussis T Lomba I Ditsch N Immunosuppressive glycodelin A is an independent marker for poor prognosis in endometrial cancer. BMC Cancer (2013) 13:616. 10.1186/1471-2407-13-616 24377825 PMC3898404

[18] Jeschke U Bischof A Speer R Briese V Richter DU Bergemann C Development of monoclonal and polyclonal antibodies and an ELISA for the determination of glycodelin in human serum, amniotic fluid and cystic fluid of benign and malignant ovarian tumors. Anticancer Res (2005) 25(3A):1581–9. 16033064

[19] Li TC Okon MA Dalton CF Heatley M Laird SM. Is the measurement of placental protein-14 and CA-125 in plasma and uterine flushings useful in the evaluation of peri-menopausal and post-menopausal bleeding? Hum Reprod (1998) 13(1O):2895–901. 10.1093/humrep/13.10.2895 9804252

[20] Kamarainen M Leivo I Koistinen R Julkunen M Karvonen U Rutanen EM Normal human ovary and ovarian tumors express glycodelin, a glycoprotein with immunosuppressive and contraceptive properties. Am J Pathol (1996) 148(5):1435–43. 8623915 PMC1861557

[21] Jeschke U Mylonas I Kunert-Keil C Stahn R Scholz C Janni W Immunohistochemistry, glycosylation and immunosuppression of glycodelin in human ovarian cancer. Histochem Cell Biol (2009) 131(2):283–95. 10.1007/s00418-008-0510-z 18853174

[22] Scholz C Heublein S Lenhard M Friese K Mayr D Jeschke U. Glycodelin A is a prognostic marker to predict poor outcome in advanced stage ovarian cancer patients. BMC Res Notes (2012) 5:551. 10.1186/1756-0500-5-551 23036050 PMC3599868

[23] Mandelin E Lassus H Seppala M Leminen A Gustafsson JA Cheng G Glycodelin in ovarian serous carcinoma: association with differentiation and survival. Cancer Res (2003) 63(19):6258–64. 14559812

[24] Bischof A Briese V Richter DU Bergemann C Friese K Jeschke U. Measurement of glycodelin A in fluids of benign ovarian cysts, borderline tumours and malignant ovarian cancer. Anticancer Res (2005) 25(3A):1639–44. 16033074

[25] Havrilesky LJ Whitehead CM Rubatt JM Cheek RL Groelke J He Q Evaluation of biomarker panels for early stage ovarian cancer detection and monitoring for disease recurrence. Gynecol Oncol (2008) 110(3):374–82. 10.1016/j.ygyno.2008.04.041 18584856

[26] Yaniv E Borovsky Z Mishan-Eisenberg G Rachmilewitz J. Placental protein 14 regulates selective B cell responses. Cell Immunol (2003) 222(2):156–63. 10.1016/S0008-8749(03)00129-1 12826085

[27] Badr IH El Sayedb HM Alc HA Hegaba MS Clinical utility of serum glycodelin as a novel marker for ovarian cancer. Life Sci J (2013) 10(3):664–70.

[28] Richter C Baetje M Bischof A Makovitzky J Richter DU Gerber B Expression of the glycodelin A gene and the detection of its protein in tissues and serum of ovarian carcinoma patients. Anticancer Res (2007) 27(4A):2023–5. 17649816

[29] Riittinen L. Serous ovarian cyst fluids contain high levels of endometrial placental protein 14. Tumour Biol (1992) 13(3):175–9. 10.1159/000217762 1626182

[30] Perwez Hussain S Harris CC. Inflammation and cancer: an ancient link with novel potentials. Int J Cancer (2007) 121(11):2373–80. 10.1002/ijc.23173 17893866

[31] Balkwill FR Re: Possible role of ovarian epithelial inflammation in ovarian cancer. J Natl Cancer Inst (1999) 92(2):162–3. 10.1093/jnci/92.2.162A 10639521

[32] Loukovaara S Immonen IR Loukovaara MJ Koistinen R Kaaja RJ. Glycodelin: a novel serum anti-inflammatory marker in type 1 diabetic retinopathy during pregnancy. Acta Ophthalmol Scand (2007) 85(1):46–9. 10.1111/j.1600-0420.2006.00766.x 17244209

[33] Tsviliana A Mayr D Kuhn C Kunze S Mylonas I Jeschke U Determination of glycodelin-A expression correlated to grading and staging in ovarian carcinoma tissue. Anticancer Res (2010) 30(5):1637–40. 20592354

[34] Hautala LC Greco D Koistinen R Heikkinen T Heikkila P Aittomaki K Glycodelin expression associates with differential tumour phenotype and outcome in sporadic and familial non-BRCA1/2 breast cancer patients. Breast Cancer Res Treat (2011) 128(1):85–95. 10.1007/s10549-010-1065-y 20676758

[35] Kostadima L Pentheroudakis G Fountzilas G Dimopoulos M Pectasides D Gogas H Survivin and glycodelin transcriptional activity in node-positive early breast cancer: mRNA expression of two key regulators of cell survival. Breast Cancer Res Treat (2006) 100(2):161–7. 10.1007/s10549-006-9240-x 16823513

[36] Shabani N Mylonas I Kunert-Keil C Briese V Janni W Gerber B Expression of glycodelin in human breast cancer: immunohistochemical analysis in mammary carcinoma in situ, invasive carcinomas and their lymph node metastases. Anticancer Res (2005) 25(3A):1761–4. 16033096

[37] Jeschke U Mylonas I Kunert-Keil C Dazert E Shabani N Werling M Expression of glycodelin protein and mRNA in human ductal breast cancer carcinoma in situ, invasive ductal carcinomas, their lymph node and distant metastases, and ductal carcinomas with recurrence. Oncol Rep (2005) 13(3):413–9. 15706409

[38] Scholz C Toth B Barthell E Mylonas I Weissenbacher T Friese K Glycodelin expression in correlation to grading, nodal involvement and steroid receptor expression in human breast cancer patients. Anticancer Res (2010) 30(5):1599–603. 20592348

[39] Kamarainen M Halttunen M Koistinen R von Boguslawsky K von Smitten K Andersson LC Expression of glycodelin in human breast and breast cancer. Int J Cancer (1999) 83(6):738–42. 10.1002/(SICI)1097-0215(19991210)83:6<738::AID-IJC7>3.0.CO;2-F 10597188

[40] Connor JP Brudney A Ferrer K Fazleabas AT. Glycodelin-A expression in the uterine cervix. Gynecol Oncol (2000) 79(2):216–9. 10.1006/gyno.2000.5941 11063647

[41] Schneider MA Granzow M Warth A Schnabel PA Thomas M Herth FJ Glycodelin: a new biomarker with immunomodulatory functions in non-small cell lung cancer. Clin Cancer Res (2015) 21(15):3529–40. 10.1158/1078-0432.CCR-14-2464 25901080

[42] Hautala L Koistinen H Abstract 601: Glycodelin expression in lung cancer and melanoma. Cancer Res (2015) 75(15 Suppl):601 10.1158/1538-7445.AM2015-601

[43] Kunert-Keil C Steinmuller F Jeschke U Gredes T Gedrange T. Immunolocalization of glycodelin in human adenocarcinoma of the lung, squamous cell carcinoma of the lung and lung metastases of colonic adenocarcinoma. Acta Histochem (2011) 113(8):798–802. 10.1016/j.acthis.2010.11.009 21168900

[44] Govindarajan R Parthasarathy S Glycodelin: a possible new biological marker in colorectal cancer. J Clin Oncol (2006) 24(18_suppl):20081 10.1200/jco.2006.24.18_suppl.20081

[45] Schneider MA Muley T Kahn NC Warth A Thomas M Herth FJ Glycodelin is a potential novel follow-up biomarker for malignant pleural mesothelioma. Oncotarget (2016) 7(44):71285–97. 10.18632/oncotarget.12474 27713145 PMC5342078

[46] Ren S Liu S Howell PM Jr Zhang G Pannell L Samant R Functional characterization of the progestagen-associated endometrial protein gene in human melanoma. J Cell Mol Med (2010) 14(6B):1432–42. 10.1111/j.1582-4934.2009.00922.x 19799645 PMC3829010

[47] Ren S Howell PM Jr Han Y Wang J Liu M Wang Y Overexpression of the progestagen-associated endometrial protein gene is associated with microphthalmia-associated transcription factor in human melanoma. Ochsner J (2011) 11(3):212–9. 21960753 PMC3179191

[48] Kamarainen M Miettinen M Seppala M von Boguslawsky K Benassi MS Bohling T Epithelial expression of glycodelin in biphasic synovial sarcomas. Int J Cancer (1998) 76(4):487–90. 10.1002/(SICI)1097-0215(19980518)76:4<487::AID-IJC7>3.0.CO;2-N 9590122

[49] Schneider MA Kahn NC Thomas M Herth FJF Muley T Heussel CP The pregnancy associated protein glycodelin as a follow-up biomarker in a male non-small cell lung cancer patient. Cancer Treat Commun (2015) 4:139–42. 10.1016/j.ctrc.2015.09.005

[50] Koistinen H Seppala M Nagy B Tapper J Knuutila S Koistinen R. Glycodelin reduces carcinoma-associated gene expression in endometrial adenocarcinoma cells. Am J Obstet Gynecol (2005) 193(6):1955–60. 10.1016/j.ajog.2005.05.073 16325596

[51] Ohta K Maruyama T Uchida H Ono M Nagashima T Arase T Glycodelin blocks progression to S phase and inhibits cell growth: a possible progesterone-induced regulator for endometrial epithelial cell growth. Mol Hum Reprod (2008) 14(1):17–22. 10.1093/molehr/gam081 18178606

[52] Kamarainen M Seppala M Virtanen I Andersson LC. Expression of glycodelin in MCF-7 breast cancer cells induces differentiation into organized acinar epithelium. Lab Invest (1997) 77(6):565–73. 9426393

[53] Hautala LC Koistinen R Seppala M Butzow R Stenman UH Laakkonen P Glycodelin reduces breast cancer xenograft growth in vivo. Int J Cancer (2008) 123(10):2279–84. 10.1002/ijc.23773 18720404

[54] Hautala LC Koistinen R Koistinen H Abstract 4066: Glycodelin abolishes PMA-induced migration of MCF-7 breast cancer cells. Cancer Res (2014) 74(19 Suppl):4066 10.1158/1538-7445.AM2014-4066

[55] Kim JG Ramachandran S Zhou HM Rayner D Parthasarathy S. Implications in the maintenance of pregnancy: I. Presence of immunoreactive glycodelin in human umbilical cord vein endothelial cells. Fertil Steril (2000) 73(4):839–42. 10.1016/S0015-0282(99)00599-3 10731550

[56] Zhou HM Ramachandran S Kim JG Raynor DB Rock JA Parthasarathy S. Implications in the management of pregnancy: II. Low levels of gene expression but enhanced uptake and accumulation of umbilical cord glycodelin. Fertil Steril (2000) 73(4):843–7. 10.1016/S0015-0282(99)00600-7 10731551

[57] Song M Ramaswamy S Ramachandran S Flowers LC Horowitz IR Rock JA Angiogenic role for glycodelin in tumorigenesis. Proc Natl Acad Sci U S A (2001) 98(16):9265–70. 10.1073/pnas.151151198 11459932 PMC55409

[58] Pala A D’Elia P Spampinato G Pittaluga E Benagiano G Human amniotic glycodelin actively regulates changes in β-catenin immunoreactivity in cultured human umbilical vein endothelial cells (HUVEC). J Matern Fetal Neonatal Med (2012) 25(8):1514–6. 10.3109/14767058.2011.629258 21999200

[59] Rajabi M Mousa SA The role of angiogenesis in cancer treatment. Biomedicines (2017) 5(2):E34 10.3390/biomedicines5020034 28635679 PMC5489820

[60] Bolton AE Pockley AG Clough KJ Mowles EA Stoker RJ Westwood OM Identification of placental protein 14 as an immunosuppressive factor in human reproduction. Lancet (1987) 1(8533):593–5. 10.1016/S0140-6736(87)90235-2 2881133

[61] Pockley AG Mowles EA Stoker RJ Westwood OM Chapman MG Bolton AE. Suppression of in vitro lymphocyte reactivity to phytohemagglutinin by placental protein 14. J Reprod Immunol (1988) 13(1):31–9. 10.1016/0165-0378(88)90046-0 3418616

[62] Pockley AG Bolton AE. Placental protein 14 (PP14) inhibits the synthesis of interleukin-2 and the release of soluble interleukin-2 receptors from phytohaemagglutinin-stimulated lymphocytes. Clin Exp Immunol (1989) 77(2):252–6. 2789118 PMC1542003

[63] Mukhopadhyay D Sundereshan S Rao C Karande AA. Placental protein 14 induces apoptosis in T cells but not in monocytes. J Biol Chem (2001) 276(30):28268–73. 10.1074/jbc.M010487200 11325960

[64] Mishan-Eisenberg G Borovsky Z Weber MC Gazit R Tykocinski ML Rachmilewitz J. Differential regulation of Th1/Th2 cytokine responses by placental protein 14. J Immunol (2004) 173(9):5524–30. 10.4049/jimmunol.173.9.5524 15494501

[65] Lee CL Chiu PC Lam KK Siu SO Chu IK Koistinen R Differential actions of glycodelin-A on Th-1 and Th-2 cells: a paracrine mechanism that could produce the Th-2 dominant environment during pregnancy. Hum Reprod (2011) 26(3):517–26. 10.1093/humrep/deq381 21227941

[66] Soni C Karande AA. Glycodelin A suppresses the cytolytic activity of CD8+ T lymphocytes. Mol Immunol (2010) 47(15):2458–66. 10.1016/j.molimm.2010.06.008 20638131

[67] Rachmilewitz J Riely GJ Tykocinski ML. Placental protein 14 functions as a direct T-cell inhibitor. Cell Immunol (1999) 191(1):26–33. 10.1006/cimm.1998.1408 9918684

[68] Rachmilewitz J Riely GJ Huang JH Chen A Tykocinski ML. A rheostatic mechanism for T-cell inhibition based on elevation of activation thresholds. Blood (2001) 98(13):3727–32. 10.1182/blood.V98.13.3727 11739178

[69] Rachmilewitz J Borovsky Z Mishan-Eisenberg G Yaniv E Riely GJ Tykocinski ML. Focal localization of placental protein 14 toward sites of TCR engagement. J Immunol (2002) 168(6):2745–50. 10.4049/jimmunol.168.6.2745 11884441

[70] Rachmilewitz J Borovsky Z Riely GJ Miller R Tykocinski ML. Negative regulation of T cell activation by placental protein 14 is mediated by the tyrosine phosphatase receptor CD45. J Biol Chem (2003) 278(16):14059–65. 10.1074/jbc.M211716200 12556471

[71] Ish-Shalom E Gargir A Andre S Borovsky Z Ochanuna Z Gabius HJ Alpha2,6-sialylation promotes binding of placental protein 14 via its Ca2+-dependent lectin activity: insights into differential effects on CD45RO and CD45RA T cells. Glycobiology (2006) 16(3):173–83. 10.1093/glycob/cwj053 16269626

[72] Soni C Karande AA. Glycodelin-A interferes with IL-2/IL-2R signalling to induce cell growth arrest, loss of effector functions and apoptosis in T-lymphocytes. Hum Reprod (2012) 27(4):1005–15. 10.1093/humrep/der477 22313865

[73] SundarRaj S Mukhopadhyay D Karande AA. Glycodelin A triggers mitochondrial stress and apoptosis in T cells by a mechanism distinct and independent of TCR signaling. Mol Immunol (2008) 45(8):2391–400. 10.1016/j.molimm.2007.11.004 18155767

[74] SundarRaj S Soni C Karande AA. Glycodelin A triggers T cell apoptosis through a novel calcium-independent galactose-binding lectin activity. Mol Immunol (2009) 46(16):3411–9. 10.1016/j.molimm.2009.07.013 19683346

[75] Jayachandran R Shaila MS Karande AA. Analysis of the role of oligosaccharides in the apoptotic activity of glycodelin A. J Biol Chem (2004) 279(10):8585–91. 10.1074/jbc.M310480200 14679202

[76] Ponnalagu D Karande AA. Mapping the apoptosis inducing domain of an immunomodulatory protein: glycodelin A. Mol Cell Biochem (2013) 377(1–2):131–41. 10.1007/s11010-013-1578-x 23392770

[77] Mukhopadhyay D SundarRaj S Alok A Karande AA. Glycodelin A, not glycodelin S, is apoptotically active. Relevance of sialic acid modification. J Biol Chem (2004) 279(10):8577–84. 10.1074/jbc.M306673200 14679205

[78] Karande AA Mukhopadhyay D Jayachandran R Sundarraj S Alok A. Mechanism of the immunomodulatory activity of glycodelin. Indian J Physiol Pharmacol (2005) 49(3):271–83. 16440844

[79] Lee CL Pang PC Yeung WS Tissot B Panico M Lao TT Effects of differential glycosylation of glycodelins on lymphocyte survival. J Biol Chem (2009) 284(22):15084–96. 10.1074/jbc.M807960200 19240032 PMC2685690

[80] Scholz C Rampf E Toth B Brunnhuber R Weissenbacher T Friese K Ovarian cancer-derived glycodelin impairs in vitro dendritic cell maturation. J Immunother (2009) 32(5):492–7. 10.1097/CJI.0b013e3181a59fa9 19609241

[81] Ren S Chai L Wang C Li C Ren Q Yang L Human malignant melanoma-derived progestagen-associated endometrial protein immunosuppresses T lymphocytes in vitro. PLoS One (2015) 10(3):e0119038. 10.1371/journal.pone.0119038 25785839 PMC4364885

[82] Scholz C Toth B Brunnhuber R Rampf E Weissenbacher T Santoso L Glycodelin A induces a tolerogenic phenotype in monocyte-derived dendritic cells in vitro. Am J Reprod Immunol (2008) 60(6):501–12. 10.1111/j.1600-0897.2008.00647.x 19032611

[83] Vigne JL Hornung D Mueller MD Taylor RN. Purification and characterization of an immunomodulatory endometrial protein, glycodelin. J Biol Chem (2001) 276(20):17101–5. 10.1074/jbc.M010451200 11278680

[84] Tee MK Vigne JL Yu J Taylor RN. Natural and recombinant human glycodelin activate a proapoptotic gene cascade in monocyte cells. J Leukoc Biol (2008) 83(4):843–52. 10.1189/jlb.0406291 18203874

[85] Miller RE Fayen JD Chakraborty S Weber MC Tykocinski ML. A receptor for the lipocalin placental protein 14 on human monocytes. FEBS Lett (1998) 436(3):455–60. 10.1016/S0014-5793(98)01184-3 9801168

[86] Alok A Mukhopadhyay D Karande AA. Glycodelin A, an immunomodulatory protein in the endometrium, inhibits proliferation and induces apoptosis in monocytic cells. Int J Biochem Cell Biol (2009) 41(5):1138–47. 10.1016/j.biocel.2008.10.009 18996219

[87] Lee CL Li K Lam KK Ng EH Koistinen H Seppala M Glycodelin-A treatment reduces the adverse effect of macrophage co-culture on human sperm motility. Mol Reprod Dev (2014) 81(6):482–3. 10.1002/mrd.22331 24733737

[88] Lee CL Lam EY Lam KK Koistinen H Seppala M Ng EH Glycodelin-A stimulates interleukin-6 secretion by human monocytes and macrophages through L-selectin and the extracellular signal-regulated kinase pathway. J Biol Chem (2012) 287(44):36999–7009. 10.1074/jbc.M112.385336 22977256 PMC3481301

[89] Koopman LA Kopcow HD Rybalov B Boyson JE Orange JS Schatz F Human decidual natural killer cells are a unique NK cell subset with immunomodulatory potential. J Exp Med (2003) 198(8):1201–12. 10.1084/jem.20030305 14568979 PMC2194228

[90] Okamoto N Uchida A Takakura K Kariya Y Kanzaki H Riittinen L Suppression by human placental protein 14 of natural killer cell activity. Am J Reprod Immunol (1991) 26(4):137–42. 10.1111/j.1600-0897.1991.tb00713.x 1840727

[91] Lee CL Chiu PC Lam KK Chan RW Chu IK Koistinen R Glycodelin-A modulates cytokine production of peripheral blood natural killer cells. Fertil Steril (2010) 94(2):769–71. 10.1016/j.fertnstert.2009.10.009 19945098

[92] Dell A Morris HR Easton RL Panico M Patankar M Oehniger S Structural analysis of the oligosaccharides derived from glycodelin, a human glycoprotein with potent immunosuppressive and contraceptive activities. J Biol Chem (1995) 270(41):24116–26. 10.1074/jbc.270.41.24116 7592613

[93] Arnold JT Kaufman DG Seppala M Lessey BA. Endometrial stromal cells regulate epithelial cell growth in vitro: a new co-culture model. Hum Reprod (2001) 16(5):836–45. 10.1093/humrep/16.5.836 11331626

[94] Xu Y Fang XJ Casey G Mills GB. Lysophospholipids activate ovarian and breast cancer cells. Biochem J (1995) 309(Pt 3):933–40. 10.1042/bj3090933 7639713 PMC1135721

[95] Xu Y Shen Z Wiper DW Wu M Morton RE Elson P Lysophosphatidic acid as a potential biomarker for ovarian and other gynecologic cancers. JAMA (1998) 280(8):719–23. 10.1001/jama.280.8.719 9728644

[96] Ramachandran S Ramaswamy S Cho C Parthasarathy S. Lysophosphatidic acid induces glycodelin gene expression in cancer cells. Cancer Lett (2002) 177(2):197–202. 10.1016/S0304-3835(01)00807-2 11825667

[97] West AC Johnstone RW. New and emerging HDAC inhibitors for cancer treatment. J Clin Invest (2014) 124(1):30–9. 10.1172/JCI69738 24382387 PMC3871231

[98] Uchida H Maruyama T Ono M Ohta K Kajitani T Masuda H Histone deacetylase inhibitors stimulate cell migration in human endometrial adenocarcinoma cells through up-regulation of glycodelin. Endocrinology (2007) 148(2):896–902. 10.1210/en.2006-0896 17068141

[99] Uchida H Maruyama T Nagashima T Asada H Yoshimura Y. Histone deacetylase inhibitors induce differentiation of human endometrial adenocarcinoma cells through up-regulation of glycodelin. Endocrinology (2005) 146(12):5365–73. 10.1210/en.2005-0359 16123169

[100] Stewart DR Erikson MS Erikson ME Nakajima ST Overstreet JW Lasley BL The role of relaxin in glycodelin secretion. J Clin Endocrinol Metab (1997) 82(3):839–46. 10.1210/jcem.82.3.3839 9062493

[101] Tseng L Zhu HH Mazella J Koistinen H Seppala M. Relaxin stimulates glycodelin mRNA and protein concentrations in human endometrial glandular epithelial cells. Mol Hum Reprod (1999) 5(4):372–5. 10.1093/molehr/5.4.372 10321810

[102] Taylor RN Vigne JL Zhang P Hoang P Lebovic DI Mueller MD. Effects of progestins and relaxin on glycodelin gene expression in human endometrial cells. Am J Obstet Gynecol (2000) 182(4):841–7; discussion 7–9. 10.1016/S0002-9378(00)70333-4 10764460

[103] Morrow DM Xiong N Getty RR Ratajczak MZ Morgan D Seppala M Hematopoietic placental protein 14. An immunosuppressive factor in cells of the megakaryocytic lineage. Am J Pathol (1994) 145(6):1485–95. 7992851 PMC1887503

[104] Kamarainen M Riittinen L Seppala M Palotie A Andersson LC Progesterone-associated endometrial protein – a constitutive marker of human erythroid precursors. Blood (1994) 84(2):467–73. 8025274

[105] Couzin-Frankel J Breakthrough of the year 2013. Cancer immunotherapy. Science (2013) 342(6165):1432–3. 10.1126/science.342.6165.1432 24357284

[106] Topalian SL Drake CG Pardoll DM. Immune checkpoint blockade: a common denominator approach to cancer therapy. Cancer Cell (2015) 27(4):450–61. 10.1016/j.ccell.2015.03.001 25858804 PMC4400238

[107] Dulos J Carven GJ van Boxtel SJ Evers S Driessen-Engels LJ Hobo W PD-1 blockade augments Th1 and Th17 and suppresses Th2 responses in peripheral blood from patients with prostate and advanced melanoma cancer. J Immunother (2012) 35(2):169–78. 10.1097/CJI.0b013e318247a4e7 22306905

[108] Curiel TJ Coukos G Zou L Alvarez X Cheng P Mottram P Specific recruitment of regulatory T cells in ovarian carcinoma fosters immune privilege and predicts reduced survival. Nat Med (2004) 10(9):942–9. 10.1038/nm1093 15322536

